# Lack of Periplasmic Non-heme Protein SorA Increases *Shewanella decolorationis* Current Generation

**DOI:** 10.3389/fmicb.2020.00262

**Published:** 2020-02-25

**Authors:** Guannan Kong, Da Song, Jun Guo, Guoping Sun, Chunjie Zhu, Fusheng Chen, Yonggang Yang, Meiying Xu

**Affiliations:** ^1^School of Biology and Biological Engineering, South China University of Technology, Guangzhou, China; ^2^Guangdong Institute of Microbiology, Guangdong Academy of Sciences, Guangzhou, China; ^3^State Key Laboratory of Applied Microbiology Southern China, Guangzhou, China; ^4^College of Food Science and Technology, Henan University of Technology, Zhengzhou, China; ^5^Guangdong Provincial Key Laboratory of Microbial Culture Collection and Application, Guangzhou, China

**Keywords:** extracellular electron transport, molybdenum-binding protein, SorA, current generation, biofilm

## Abstract

Bacterial extracellular electron transport (EET) plays an important role in many natural and engineering processes. Some periplasmic non-heme redox proteins usually coexist with *c*-type cytochromes (CTCs) during the EET process. However, in contrast to CTCs, little is known about the roles of these non-heme redox proteins in EET. In this study, the transcriptome of *Shewanella decolorationis* S12 showed that the gene encoding a periplasmic sulfite dehydrogenase molybdenum-binding subunit SorA was significantly up-regulated during electrode respiration in microbial fuel cells (MFCs) compared with that during azo-dye reduction. The maximum current density of MFCs catalyzed by a mutant strain lacking SorA (Δ*sorA*) was 25% higher than that of wild strain S12 (20 vs. 16 μA/cm^2^). Both biofilm formation and the current generation of the anodic biofilms were increased by the disruption of *sorA*, which suggests that the existence of SorA in *S. decolorationis* S12 inhibits electrode respiration. In contrast, disruption of *sorA* had no effect on respiration by *S. decolorationis* S12 with oxygen, fumarate, azo dye, or ferric citrate as electron acceptors. This is the first report of the specific effect of a periplasmic non-heme redox protein on EET to electrode and provides novel information for enhancing bacterial current generation.

## Introduction

Bacterial extracellular electron transport (EET) exists widely in natural environments. EET is an important driving force in the biogeochemical cycle of many elements and plays important roles in bio-electrochemical and bioremediation processes. In recent years, many important discoveries have been made in the field of EET, such as EET based on nanowires (Reguera et al., 2005; Ueki et al., 2018; Wang et al., 2019), and EET between different microbial cells (Summers et al., 2010).

*Shewanella* and *Geobacter* have been used as the main model organisms in EET studies. The membrane-spanning *c*-type cytochrome (CTC) system plays an essential role in the EET of both *Geobacter* and *Shewanella* (Xiao et al., 2012; Yang et al., 2012). Compared with the outer membrane proteins, much more redox proteins, including CTC and non-CTC proteins, locate within the periplasmic space and form a complex periplasmic electric network. The interactions among these periplasmic proteins are important to EET. For example, CymA and MtrA are key CTCs in the EET pathway of *Shewanella oneidensis* MR-1. However, it has been assumed that the periplasm space is too wide (23.5 ± 3.7 nm) to allow direct electron transfer from CymA to MtrA. Additional redox proteins are needed to mediate electrons between them (Dohnalkova et al., 2011; Sturm et al., 2015). STC and FccA in the periplasmic space could bind CymA and MtrA, respectively, but still cannot fill the gap between CymA and MtrA (Coursolle and Gralnick, 2010; Fonseca et al., 2013). The mutant strain lacking either STC or FccA has a minor effect on the EET capability compared to wild strain *S. oneidensis* MR-1 (Bretschger et al., 2007; Schuetz et al., 2009; Firer-Sherwood et al., 2011), and a double-deletion mutant (lacking both STC and FccA) also showed alternative pathways for EET with a lag-phase of several hours (Sturm et al., 2015), indicating that more redox proteins are involved in periplasmic electron transfer.

The co-existing redox proteins in the periplasm may have different effects on the EET pathway, i.e., facilitating or suppressing EET. In addition to CTC, some alternative redox proteins that participate in or facilitate EET have been found in both Gram-negative and Gram-positive bacteria, such as the periplasmic sulfide reductase Psr in *S. oneidensis* MR-1 and the peptidoglycan-associated lipoprotein PplA in *Listeria monocytogenes* (Jormakka et al., 2008; Zhang et al., 2014; Kondo et al., 2015; Light et al., 2018). However, information on the EET-suppressive proteins or pathways in bacteria is lacking.

In this study, we found that the transcription levels of four genes of *Shewanella decolorationis* S12, *SHD2782–SHD2785*, were significantly up-regulated during EET to an electrode, compared that in anaerobic respiration with the azo-dye amaranth. Gene *SHD2784* encodes a periplasmic sulfite dehydrogenase molybdenum-binding subunit SorA. The disruption of this gene resulted in an increase of 25% in the maximum current density compared to the wild strain but had no effect on the reduction of azo dye and Fe (III) citrate. The results indicate a specific role of this gene in electrode respiration of *S. decolorationis* S12. This study provides novel information to understand the EET of *Shewanella* species and new insight into strategies for enhancing the efficiency of EET.

## Materials and Methods

### Bacterial Strains, Plasmids, and Growth Conditions

A list of all bacterial strains and plasmids used in this study is given in Table 1. *S. decolorationis* S12 was isolated from the activated sludge of a textile-wastewater treatment plant (Xu et al., 2005). S12 mutants and complemented strains were constructed using the methods described previously (Fang et al., 2019). *Escherichia coli and S. decolorationis* strains were cultured aerobically in LB medium at 37 and 30°C, respectively. Plasmid pHGM01 was used to construct the mutant strains with Ap^r^, Gm^r^, Cm^r^, and *att*-based suicide vector. Plasmid pHG102 was used to construct the complemented strains with Km^r^ and the *S. oneidensis arcA* promoter vector (Fang et al., 2019). Gentamycin (15 μg/mL), ampicillin (50 μg/mL), or 2,6-diaminopimelic acid (30 μM) were added to the medium as necessary.

**TABLE 1:** Strains and plasmids used in this study.

Strain or plasmid	Description	Reference or source
***E. coli* strain**		
WM3064	Host for *pir*-dependent plasmids and donor strain for conjugation; Δ*dapA*	Fang et al., 2019
*S. decolorationis* S12	Wild type	Lab stock
Δ*SHD2782* (Δ*mcc*)	S12 mutant with gene SHD2782 deleted	Kong et al., 2017
Δ*SHD2783* (Δ*sorB*)	S12 mutant with gene *SHD2783* deleted	This study
Δ*SHD2784* (Δ*sorA)*	S12 mutant with gene *SHD2784* deleted	This study
*SHD2784*^*c*^(Δ*sorA^*c*^)*	SorA complemented strain	This study
Δ*SHD2785*	S12 mutant with gene *SHD2785* deleted	This study
Δ*mcc-sorA-sorB*	S12 mutant with genes SHD2782, SHD2783, and SHD2784 deleted	This study
Δ*mtrC-omcA*	S12 mutant with genes *mtrC* and *omcA*	This study
**Plasmid**		
pHGM01	Ap^r^, Gm^r^, Cm^r^, *att*-based suicide vector	Fang et al., 2019
pHG102	Broad-host Km^r^ vector containing the *S. oneidensis arcA* promoter	Fang et al., 2019

### Bacteria Cultivation

*Shewanella decolorationis* S12 was cultivated by transferring a single clone to a 250-mL conical flask containing 100 mL LB medium (10 g/L peptone, 5 g/L yeast extract, 5 g/L NaCl) and was then incubated in a shaker (120 r/min, 30°C) overnight. The cells were harvested in the middle of the exponential growth phase (approximately 12 h) by centrifugation, washed twice, and re-suspended with phosphate buffer (0.1 M, pH 7.2) prior to inoculation. The cells were inoculated into the lactate mineral (LM) medium [12.8 g/L of Na_2_HPO_4_, 3 g/L of KH_2_PO_4_, 0.5 g/L of NaCl, 1.0 g/L of NH_4_Cl, 0.05% (w/v) yeast extract, lactate 2.5 mM, and 10 mM fumarate, pH 6.8] at a final OD_600_ of 0.04. The medium was then flushed with nitrogen for 15 min to achieve anaerobic conditions and statically cultivated at 30°C in an anaerobic workstation (BugBox, Ruskinn Technologies). All batch experiments were conducted in 100-mL serum bottles containing 40 mL LM medium. In each aerobic cultivation flask or anaerobic serum bottle, one piece of graphite plate (1 cm × 3 cm × 0.2 cm) was stabilized under the culture liquid with a titanium wire to evaluate the biofilm growth. All cultures were prepared in triplicate.

### Microbial Fuel Cell (MFC) Assembly and Operation

Dual-chamber glass microbial fuel cells (MFCs) were assembled as previously described (Yang et al., 2014). Briefly, plain graphite plates (2 cm × 3 cm × 0.2 cm) were used as anodes and cathodes. An Ag/AgCl electrode [0.197 V vs. standard hydrogen electrode (SHE)] was used as a reference electrode to each anode. The anode and cathode chambers were separated with a piece of Nafion115 membrane (7.1 cm^2^). After assembly and sterilization (115°C for 20 min), the anode chamber (120 mL) was filled with 100 mL of LM (pH 6.8). To stimulate biofilm growth, 0.05% (w/v) yeast extract was added to the medium (Lanthier et al., 2008). The total sulfur concentration in the medium was 0.25 mg/L due to the addition of yeast extract. Each cathode chamber was filled with 100 mL sterilized phosphate buffered saline (PBS) solution (pH 7.2) containing 50 mM potassium ferricyanide.

Cells in the late-exponential growth phase in LB medium were washed twice with sterilized PBS (pH 7.2) and inoculated into the medium in an anode chamber at a final OD_600_ of 0.04. The medium was then flushed with nitrogen for 15 min to achieve an anaerobic condition. The anode and cathode were connected by a titanium wire with a 1000 Ω resistor. MFCs were operated at 30°C, and all cultures were prepared in triplicate. The current of MFCs under a closed circuit condition was recorded with a multimeter (Keithley 2700, module 7702). The coulombic efficiency (CE) of the MFCs was calculated as reported previously (Ringeisen et al., 2006; Bretschger et al., 2010). Briefly, CE was calculated as *C_*e*_/C_*t*_*. *C*_*e*_ is the total coulombs contributed to current generation and was calculated by integrating each *i–t* curve with respect to time. *C*_*t*_ is the theoretical coulombic yield and was calculated as *C*_*t*_ = *FbC*, where *F* is Faraday’s constant (96,485 C/mol), *b* is the number of moles of electrons produced by per mole of substrate (4 for lactate oxidization to acetate) (Ringeisen et al., 2006; Lanthier et al., 2008), and *C* is the decrease in substrate concentration (mol, lactate) in the anodic medium. Cyclic voltammetry (CV) analysis of the MFC anodes was conducted in MFCs with the anode as the working electrode, cathode as the counter electrode, and Ag/AgCl as a reference electrode when the MFCs were operated for 48 h. The potential scan rate was 10 mV/s, as previously reported (Yang et al., 2014). A mutant strain (Δ*mtrC*&*omcA*) of S12 lacking both OmcA and MtrC was used, and LM media containing 2 μM of riboflavin and flavin mononucleotide, respectively, were used as controls to determine the redox potentials of the outer membrane CTCs and flavins, respectively.

### Chemical Analyses of the Flavins and Total Sulfur

To determine the concentration of electron mediator (i.e., flavins), 2 mL of the culture liquid was filtered via a 0.22 μm polytetrafluoroethylene filter and then analyzed by high-performance liquid chromatography (LC-20A, SHIMADZU) with a fluorescence detector (RF-10AXL, SHIMADZU). The excitation wavelength was 450 nm and the emission wavelength was 520 nm. The total sulfur content in the LM was determined by a spectrophotometric method (Dedov et al., 2018).

### Iron and Amaranth Reduction Assays

Iron and amaranth reduction experiments were conducted in serum bottles with 100 mL of LM. Sodium lactate (2.5 mM) was added as the electron donor and amaranth red (0.5 mM) or ferric citrate (4 mM) as the electron acceptor. After inoculation, the serum bottles were flushed with nitrogen for 8 min and then sealed with rubber pads. They were incubated in the anaerobic workstation (RuskinnC0105) at 30°C. All cultures were prepared in triplicate. The reduction process was analyzed by periodic sampling using disposable syringes on a clean bench.

Ferric ion concentration was determined by ferrozine assay (Lovley and Phillips, 1987). The absorption value of azo dye amaranth was analyzed by UV/Vis spectrophotometer at 520 nm (Fang et al., 2019).

### Bacterial Growth and Total-Cell Protein Determination

Aerobic growth curves of strain S12, mutants, and complemented strains were determined with an automatic growth curve analyzer (Bioscreen C). For the anaerobic cultivation with fumarate or electrode as electron acceptors, the bacteria density in the liquid culture was measured by UV–visible spectrophotometer at a wavelength of 600 nm (OD_600_). Moreover, the total-cell protein was evaluated with a Bradford Coomassie Brilliant Blue assay, as reported previously (Fang et al., 2015). All assays were performed in triplicate.

### Biofilm Observation

The biofilm was stained with a Live/Dead BacLight staining kit (Life Technologies, L7012) and observed by a confocal laser scanning microscope (CLSM) (Yang et al., 2014). All assays were performed in triplicate.

### Transcriptional Analysis

The transcriptomes of strain S12 respiring with electrode and amaranth were comparatively analyzed as described previously (Lian et al., 2016). Raw sequence data were filtered to remove the adapter sequence, reads with more than 10% unknown nucleotides, and reads with more than 50% low-quality bases (quality score ≤ 20). Clean reads were mapped into the reference genome using Tophat 2.0.1 and Samtools 0.1.18.0. Less than three mismatch bases were permitted, and unique mapped reads were obtained. Cufflinks 2.0.0 software was used for calculating the FPKM value (fragments per kilobase per million mapped reads). Differentially expressed genes were identified with | log_2_fold change| ≥ 1 and false discovery rate (FDR) < 0.05. Genes were annotated in the Cluster of Orthologous Groups (COG) of proteins database for identification of orthologous proteins and the Kyoto Encyclopedia of Genes and Genomes (KEGG) database for annotation and metabolic pathway analysis. The locations of encoded proteins were predicted using Cell-PLoc 2.0 with Gneg-mPLoc (Chou and Shen, 2010).

## Results and Discussion

### Different Transcriptomic Profiles Between Electrode and Amaranth Respiration

**FIGURE 1:**
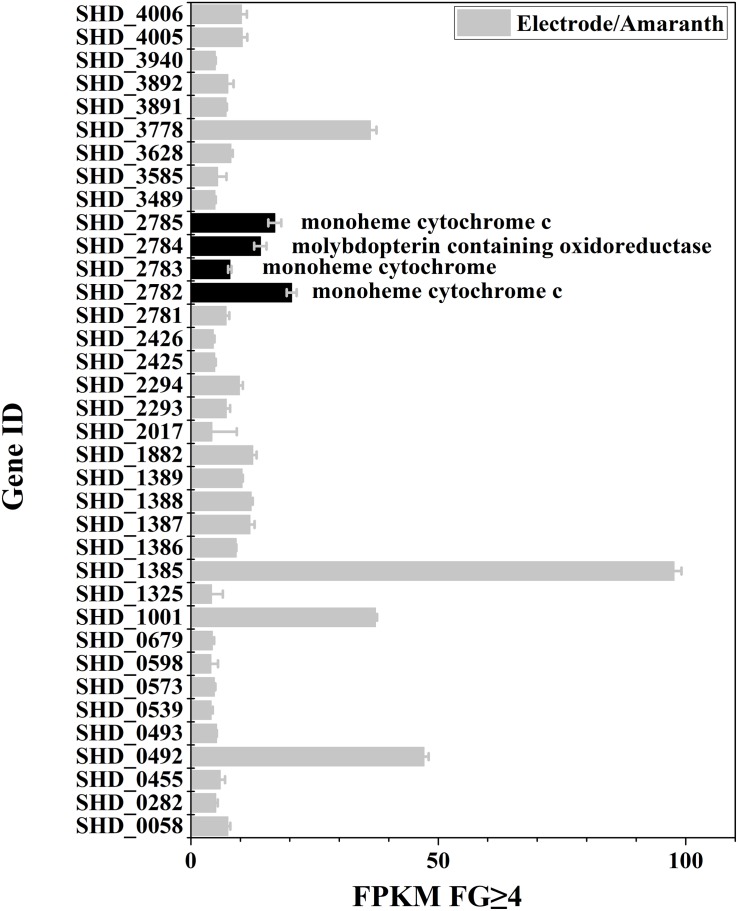
Genes with more than fourfold expression increase (FPKM-reads) during electrode-respiration vs. azo-dye respiration.

In order to find proteins that are possibly specific to different extracellular electron acceptors, transcriptomes of *S. decolorationis* S12 respiring solid graphite electrode and soluble azo dye amaranth were compared. A total of 339 genes were differentially transcribed between the electrode and amaranth respiration of strain S12, of which 36 genes were up-regulated by more than fourfolds during respiring with electrode (Figure 1 and Supplementary Table S1). Protein products of the 36 genes include secreted proteins, signal transducers, bi-component regulatory proteins, cytochrome proteins, and some hypothetical proteins. The main CTC pathway of CymA-MtrABC in EET of *S. decolorationis* S12 had no significant change between electrode and amaranth respiration.

The genes of *SHD0492*, *SHD1001*, *SHD3778*, and *SHD1385* were up-regulated by over 32-fold under the electrode respiration condition compared to that under azo-dye reduction. The genes encode for two hypothetical proteins (SHD3778 and SHD0492), a bifunctional autotransporter (SHD1385), and an exporter protein (SHD1001). Notably, four genes *SHD2782-2785*, which belong to the same gene cluster (Supplementary Figure S1A), were up-regulated by over sixfold under the electrode respiration condition, which indicated important and specific roles of this gene cluster in the electrode growth by *S. decolorationis* S12. The four genes encode monoheme CTC Mcc (SHD2782), sulfite dehydrogenase CTC subunit SorB (SHD2783), sulfite dehydrogenase molybdopterin-binding subunit SorA (SHD2784), and monoheme cytochrome c4 (SHD2785), respectively. Each protein encoded by this operon contains one heme domain (CXXCH) (Meyer et al., 2004), except for SorA (Supplementary Figure S1B), indicating that SorA is not a CTC. Among the 210 *Shewanella* genomes in NCBI, this operon was only found in 14 genomes^1^ (Supplementary Table S2). The four proteins were predicted to be located in periplasmic space by using online software^2^, which is consistent with the SorAB location in several other bacteria such as *Starkeya novella* and *Aeropyrum pernix* (Meyer et al., 2004; Denger et al., 2008). SorA often coexists with SorB for sulfite respiration (Myers and Kelly, 2005) and is predicted to have electronic interactions with trimethylamine *N*-oxide reductase (TorA) and nitrate ammonification protein (NapA) in *S. oneidensis* MR-1 (Ding et al., 2014). Our previous results showed that deletion of gene *SHD2782* in *S. decolorationis* S12 resulted in 30% decrease in current generation (Kong et al., 2017). However, the role of the other three genes (*SHD2783-2785*) in *S. decolorationis* S12 is still unknown.

### Roles of SorA in Current Generation

**FIGURE 2:**
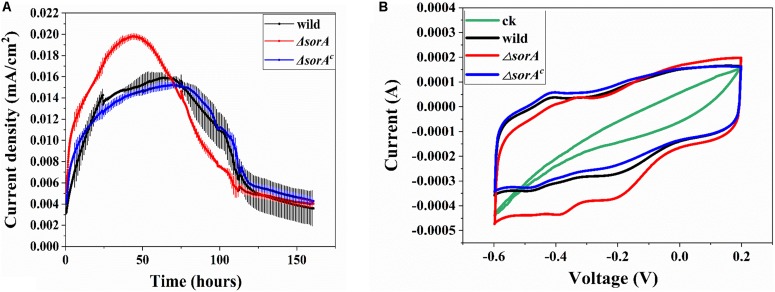
Effects of disruption in SorA on the electrochemical behavior of S12. **(A)** Current generation of mutant strain Δ*sorA* and wild strain. **(B)** CV of mutant strain Δ*sorA* and wild strain biofilms.

To investigate the role of SorA in EET to electrode, the gene *sorA*, as well as the other genes in the same operon, were knocked out. The current generation in MFCs by the wild strain and the mutants were compared. As shown in Figure 2A, the maximum current (0.02 mA/cm^2^) generated by Δ*sorA* was 1.25-fold that generated by the wild strain (0.016 mA/cm^2^) and Δ*sorA*^*c*^ (0.015 mA/cm^2^), suggesting that the presence of SorA in *S. decoloartionis* S12 inhibits the EET to electrodes. The CE of the MFCs catalyzed by the wild strain, Δ*sorA*, and Δ*sorA*^*c*^ were comparable (26.7, 24.7, and 25.3%, respectively) and also comparable to that of other *Shewanella* species (15–35%) (Bretschger et al., 2010). The CV test showed results consistent with the current-generation profiles in MFCs in that, compared with the wild and complemented strain, Δ*sorA* biofilms generated a higher current when the anode potential varied between −0.6 and 0.2 V (vs. Ag/AgCl) (Figure 2B). Two pairs of redox peaks were observed in the CV profiles of the anodic biofilms. The reductive peak at -0.18 V (vs. Ag/AgCl) can be attributed to the outer-membrane CTCs as this peak disappearing in the CV curve of the mutant strain lacking two key outer-membrane CTCs, OmcA and MtrC (Supplementary Figure S2). The reductive peak around -0.4 V (vs. Ag/AgCl) is consistent with the reduction of flavins, as this peak became higher with the addition of flavins to the anodic culture (Supplementary Figure S2). The CV profile was also consistent with that of *S. oneidensis* MR-1 (Baron et al., 2009). In contrast to Δ*sorA*, Δ*sorB* and Δ*SHD2785* generated a similar current to the wild strain (Supplementary Figure S3A). Further experiments with a multi-gene mutant (Δ*mcc-sorA-sorB*) also generated a similar current to that of the wild strain (Supplementary Figure S3B). These results suggested that genes in the operon *SHD2782-2785* have different influences on the current generation of *S. decolorationis* S12, i.e., gene *mcc* (Kong et al., 2017) can facilitate current generation, while *sorA* can inhibit it, and gene *sorB* and *SHD2785* had no influence on current generation.

**FIGURE 3:**
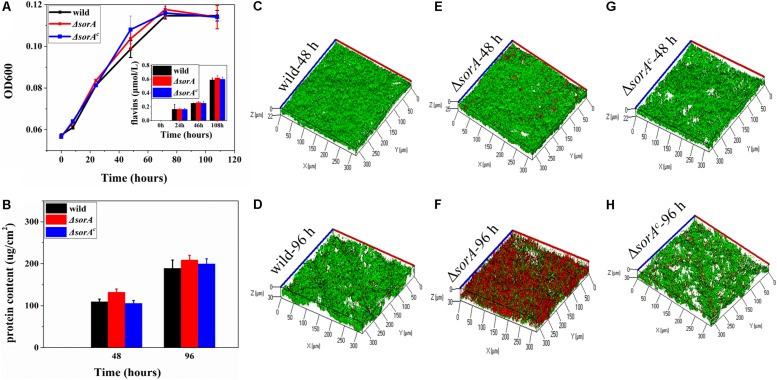
Growth of the planktonic and biofilm cells of mutant and wild strain. **(A)** Growth of planktonic cells and production of flavins in anode chamber. **(B)** Protein content of the anode biofilms. **(C)** 48-h biofilm of wild strain. **(D)** 96-h biofilm of wild strain. **(E)** 48-h biofilm of mutant strain Δ*sorA*. **(F)** 96-h biofilm of mutant strain ΔsorA. **(G)** 48-h biofilm of complemented strain Δ*sorA*^*c*^. **(H)** 96-h biofilm of complemented strain Δ*sorA*^*c*^.

To further understand how SorA affected the current generation of strain S12, the protein content of planktonic cells and the concentration of electron shuttle flavins were determined. There was no significant difference in the growth of the planktonic cells in MFCs between strain S12, mutant Δ*sorA*, and complemented strain Δ*sorA*^*c*^ (Figure 3A). The concentrations of electron shuttles (riboflavins and flavin mononucleotide), which play essential roles in the EET of *Shewanella* strains (Marsili et al., 2008), were also similar between these strains (Figure 3A). Moreover, our previous results have shown that, compared to the biofilms, planktonic cells contribute only a minor fraction (<20%) to the current generation by *S. decolorationis* S12 MFCs (Yang et al., 2014). Therefore, growth and metabolism of the planktonic cells did not contribute to the difference in current generation between the mutant Δ*sorA* and wild-type strain.

In contrast, the protein content and structures of anode biofilms were significantly different between mutant Δ*sor*A and wild-type strain, while the biofilms of complemented Δ*sorA*^*c*^ were similar to the wild-type strain (Figures 3B–H). During the current-increasing stage (48 h) in MFCs, the mutant Δ*sorA* biofilms were dense and uniform, with an average thickness of 25 μm on the anode surfaces, while the biofilm thicknesses of wild type strain S12 and complemented strain were 21.5 and 20.2 μm. Consistently, protein quantification showed that the protein density of Δ*sorA* biofilms was 128.4 μg/cm^2^, which was higher than that of the wild-type biofilms (104.3 μg/cm^2^) and the complemented strain biofilms (102.8 μg/cm^2^) (Figure 3B). In addition, as shown in Figures 3C,E,G, the viability of Δ*sorA* biofilms was comparable with that of the wild type strain and the complemented strain (90.2, 87.6, and 85.2%, respectively) during the current-increase stage. Since biofilms play a dominant role in current generation by S12 strains (Yang et al., 2014), the higher protein content and viability of Δ*sorA* biofilms could be considered a major reason for its higher current generation. It should be noted that more cells in Δ*sorA* biofilm became red (i.e., low viability) in the current-decrease stage (96 h, Figures 3D,F,H), probably because the current density had decreased to a lower level than that of the wild strain at this time, and the thicker architecture of Δ*sorA* biofilm caused more biofilm cells to be inaccessible to either electron donors (for cells at the bottom) or electron acceptors (for cells at the top). Our previous results have shown that *S. decolorationis* S12 biofilm viability could be recovered with the recovery of current generation (Yang et al., 2014).

In addition to comparing the total current generation of the MFCs catalyzed by mutant and wild-type strains, another concern was whether the disruption of *sorA* affected EET capability per g-protein. By normalizing the power density to the biofilm protein density at hour 48, it can be calculated that EET capability of wild strain is 0.134 μA/μg biofilm protein [(0.014 mA/cm^2^)/(104.3 μg/cm^2^)], while that of Δ*sorA* is 0.154 μA/μg biofilm protein [(0.02 mA/cm^2^)/(128.4 μg/cm^2^)], and it can be seen that the EET capability was enhanced by 14.9% by deleting SorA. The result indicated that the disruption of *sorA* not only promoted biofilm formation but also improved the current generation capability compared to wild type strain and complemented strain.

It can be seen that the roles of *sorA* and *mcc* are different, although they are in the same gene cluster. Mcc is a mono-heme CTC (Kong et al., 2017), and it is likely that Mcc can facilitate EET via heme–heme interaction, while SorA seems to play an inhibitory role in EET. Several reports have shown that some redundant pathways could inhibit bacterial EET (Coursolle and Gralnick, 2010; Yong et al., 2012). For example, deleting the lactate dehydrogenase Ldh in *E. coli* can increase its current generation (Yong et al., 2012). EET-capable bacteria usually have more complex electron transfer networks and an especially large number of CTCs, which may aid them in surviving in unstable environments. However, maintaining such an electron transfer system will cost extra energy in a certain environment. For example, *Geobacter sulfurreducens* uses the CbcL-pathway and ImcH-pathway for respiring with electron acceptors with high and low redox potentials, respectively. The disruption of the unnecessary CbcL-pathway under low-potential conditions can stimulate cell growth compared to the wild strain possessing both pathways (Levar et al., 2017). In *S. decolorationis* S12 periplasm, SorA co-exists with many other redox proteins, including those involved in EET. It is possible that SorA diverts electrons from the efficient EET pathways (e.g., cymA-MtrABC) to other electron pools (e.g., TorA and NapA) in the periplasm (Ding et al., 2014) and thus suppresses the current generation of strain S12.

### Effects of SorA on Other Types of Respirations by *S. decolorationis* S12

SorAB participate in the reaction of oxidizing sulfite into sulfate in some bacteria (Denger et al., 2008). However, the possible role of SorAB in *Shewanella* species remains unknown. To investigate whether SorA participated in the sulfite oxidation of sodium thiosulfate, sulfur, and sulfide of *S. decolorationis* S12, the wild strain and mutant Δ*sorA* were grown aerobically with thiosulfate, sulfur, and sulfide as the sole electron donor, respectively. The concentrations of sulfate, as the product of thiosulfate oxidization, sulfur oxidization, and sulfide oxidization generated by these strains were similar (Supplementary Figure S4), suggesting that SorA was unnecessary for sulfide oxidization by *S. decolorationis* S12. There are likely alternative pathways that might contribute to the sulfide oxidization in *S. decolorationis* S12.

**FIGURE 4:**
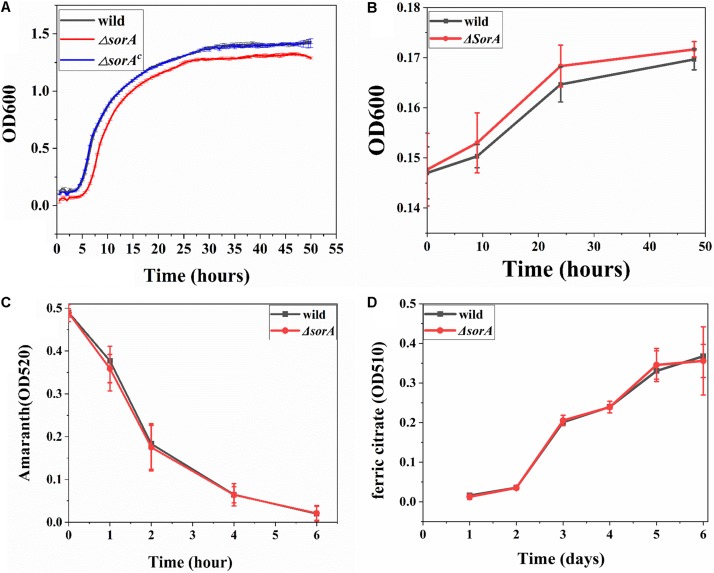
**(A)** Growth curves of planktonic cells under aerobic conditions. **(B)** Growth curves of planktonic cells respiring with fumarate. **(C)** Azo dye amaranth reduction by mutant Δ*sorA* and wild strain. **(D)** Ferric citrate reduction by mutant Δ*sorA* and wild strain.

To further understand the possible roles of SorA in other respiratory processes by *S. decolorationis* S12, the growth of Δ*sorA* with oxygen, fumarate, and two extracellular electron acceptors [azo dye amaranth and Fe (III) citrate] were tested. In aerobic and anaerobic respiration with fumarate as the electron acceptor, the planktonic cells of mutant Δ*sorA* showed a similar growth profile to that of the wild type strain, suggesting no significant effect of SorA disruption on growth development under those conditions (Figures 4A,B). Meanwhile, the biofilm structure and protein content of the mutant and wild strain on graphite plates were also similar (Supplementary Figure S5), suggesting that disruption of SorA has no direct effect on the biofilm growth of *S. decolorationis* S12 respiring with oxygen or fumarate. The results also indicate that the differences between the biofilms in Δ*sorA* MFCs and wild strain MFCs were caused by the higher current generation capability of Δ*sorA* compared to the wild strain.

Figures 4C,D show that there was no significant difference in the reduction rates of ferric citrate and amaranth between the wild and mutant strain, indicating that the gene *sor*A did not participate in iron and amaranth reduction, i.e., SorA has a specific role in EET to electrode. The electrode-specific effect of SorA is further evidence that the bacterial electron transfer strategy varies when respiring with different electron acceptors. It has been reported that *G. sulfurreducens* uses OmcB to reduce soluble or solid iron but not to reduce electrode and that *S. oneidensis* MR-1 uses OmcA to reduce electrode and Mn oxide but not to reduce ferric citrate (Richter et al., 2012). Recent studies evidenced that electron acceptor redox potential is a key variable in determining the substrate metabolism and electron transfer pathways for both *Shewanella* and *Geobacter* species (Lian et al., 2016; Levar et al., 2017; Hirose et al., 2018). In addition, some other factors such as energy generation, electron donors, and terminal reductase properties should be considered to understand the electron acceptor-specific EET strategies of bacteria further.

## Conclusion

In summary, this study evidenced an inhibitory role of the periplasmic non-heme redox protein SorA on EET to an electrode by *S. decolorationis* S12. Moreover, the effect of SorA on electron transfer seems to be electrode-specific, as the disruption of this gene has no significant effect in respiration with oxygen, fumarate, amaranth, and Fe (III) citrate as electron acceptors. This is the first report about the role of gene *sorA* in *S. decolorationis S12*. The results also provide novel information to understand the periplasmic electron transfer network of *S. decolorationis S12*.

## Data Availability Statement

The datasets analyzed in this article are not publicly available. Requests to access the datasets should be directed to YY.

## Author Contributions

All authors conceived the study and revised the manuscript. YY and MX designed the experiments. GK and CZ performed the experiments. GK, DS, FC, and YY analyzed data. GK and YY wrote the manuscript.

## Conflict of Interest

The authors declare that the research was conducted in the absence of any commercial or financial relationships that could be construed as a potential conflict of interest.

## Footnotes

1 https://www.ncbi.nlm.nih.gov/

2 http://www.csbio.sjtu.edu.cn/bioinf/Gneg-multi/

## References

[B1] Baron D. LaBelle E. Coursolle D. Gralnick J. A. Bond D. R. (2009). Electrochemical measurement of electron transfer kinetics by *Shewanella oneidensis* MR-1. *J. Biol. Chem.* 284 28865–28873. 10.1074/jbc.M109.043455 19661057 PMC2781432

[B2] Bretschger O. Cheung A. C. M. Mansfeld F. Nealson K. H. (2010). Comparative microbial fuel cell evaluations of *Shewanella* spp. *Electroanalysis* 22 883–894. 10.1002/elan.200800016

[B3] Bretschger O. Obraztsova A. Sturm C. A. Chang I. S. Gorby Y. A. Reed S. B. (2007). Current production and metal oxide reduction by *Shewanella oneidensis* MR-1 wild type and mutants. *Appl. Environ. Microbiol.* 73 7003–7012. 10.1128/AEM.01087-07 17644630 PMC2074945

[B4] Chou K. C. Shen H. B. (2010). Cell-PLoc 2.0: an improved package of web-servers for predicting subcellular localization of proteins in various organisms. *Development* 109 1091–1103. 10.4236/ns.2010.210136 18274516

[B5] Coursolle D. Gralnick J. A. (2010). Modularity of the Mtr respiratory pathway of *Shewanella oneidensis* strain MR-1. *Mol. Microbiol.* 77 995–1008. 10.1111/j.1365-2958.2010.07266.x 20598084

[B6] Dedov A. G. Marchenko D. Y. Zrelova L. V. Ivanova E. A. Sandzhieva D. A. Parkhomenko A. A. (2018). New method for determination of total of organic sulfur compounds in hydrocarbon media. *Pet. Chem.* 58 714–720. 10.1134/S0965544118080030

[B7] Denger K. Weinitschke S. Smits T. H. Schleheck D. Cook A. M. (2008). Bacterial sulfite dehydrogenases in organotrophic metabolism: separation and identification in *Cupriavidus necator* H16 and in *Delftia acidovorans* SPH-1. *Microbiology* 154 256–263. 10.1099/mic.0.2007/011650-0 18174144

[B8] Ding D. W. Xu J. Li L. Xie J. M. Sun X. (2014). Identifying the potential extracellular electron transfer pathways from a *c*-type cytochrome network. *Mol. Biosyst.* 10 3138–3146. 10.1039/C4MB00386A 25227320

[B9] Dohnalkova A. C. Marshall M. J. Arey B. W. Williams K. H. Buck E. C. Fredrickson J. K. (2011). Imaging hydrated microbial extracellular polymers: comparative analysis by electron microscopy. *Appl. Environ. Microbiol.* 77 1254–1262. 10.1128/AEM.02001-10 21169451 PMC3067245

[B10] Fang Y. Liu J. Kong G. Liu X. Yang Y. Li E. (2019). Adaptive responses of *Shewanella decolorationis* to the toxic organic extracellular electron acceptor in anaerobic respiration. *Appl. Environ. Microbiol.* 85:e00550-19. 10.1128/AEM.00550-19 31175185 PMC6677847

[B11] Fang Y. Xu M. Wu W. Chen X. Sun G. Guo J. (2015). Characterization of the enhancement of zero valent iron on microbial azo reduction. *BMC Microbiol.* 15:85. 10.1186/s12866-015-0419-3 25888062 PMC4428006

[B12] Firer-Sherwood M. A. Bewley K. D. Mock J. Y. Elliott S. J. (2011). Tools for resolving complexity in the electron transfer networks of multiheme cytochromes c. *Metallomics* 3 344–348. 10.1039/c0mt00097c 21327265

[B13] Fonseca B. M. Paquete C. M. Neto S. E. Pacheco I. Soares C. M. Louro R. O. (2013). Mind the gap: cytochrome interactions reveal electron pathways across the periplasm of *Shewanella oneidensis* MR-1. *Biochem. J.* 449 101–108. 10.1042/BJ20121467 23067389

[B14] Hirose A. Kasai T. Aoki M. Umemura T. Watanabe K. Kouzuma A. (2018). Electrochemically active bacteria sense electrode potentials for regulating catabolic pathways. *Nat. Commun.* 9:1083. 10.1038/s41467-018-03416-4 29540717 PMC5852097

[B15] Jormakka M. Yokoyama K. Yano T. Tamakoshi M. Akimoto S. Shimamura T. (2008). Molecular mechanism of energy conservation in polysulfide respiration. *Nat. Struct. Mol. Biol.* 15 730–737. 10.1038/nsmb.1434 18536726 PMC2887006

[B16] Kondo K. Okamoto A. Hashimoto K. Nakamura R. (2015). Sulfur-mediated electron shuttling sustains microbial long-distance extracellular electron transfer with the aid of metallic iron sulfides. *Langmuir* 31 7427–7434. 10.1021/acs.langmuir.5b01033 26070345

[B17] Kong G. N. Xu M. Y. Song D. Yang Y. G. (2017). Role of Mcc in *Shewanella decolorationis* S12 electrode respiration. *Microbiol. China* 44 1547–1554. 10.13344/j.microbiol.china.170072

[B18] Lanthier M. Gregory K. B. Lovley D. R. (2008). Growth with high planktonic biomass in *Shewanella oneidensis* fuel cells. *FEMS Microbiol. Lett.* 278 29–35. 17995953 10.1111/j.1574-6968.2007.00964.x PMC2228398

[B19] Levar C. E. Hoffman C. L. Dunshee A. J. Toner B. M. Bond D. R. (2017). Redox potential as a master variable controlling pathways of metal reduction by *Geobacter sulfurreducens*. *ISME J.* 11 741–752. 10.1038/ismej.2016.146 28045456 PMC5322298

[B20] Lian Y. Yang Y. Guo J. Wang Y. Li X. Fang Y. (2016). Electron acceptor redox potential globally regulates transcriptomic profiling in *Shewanella decolorationis* S12. *Sci. Rep.* 6:31143. 10.1038/srep31143 27503002 PMC4977559

[B21] Light S. H. Su L. Rivera-Lugo R. Cornejo J. A. Louie A. Iavarone A. T. (2018). A flavin-based extracellular electron transfer mechanism in diverse Gram-positive bacteria. *Nature* 562 140–144. 10.1038/s41586-018-0498-z 30209391 PMC6221200

[B22] Lovley D. R. Phillips E. J. P. (1987). Rapid assay for microbially reducible ferric iron in aquatic sediments. *Appl. Environ. Microbiol.* 53 1536–1540. 10.1128/aem.53.7.1536-1540.1987 16347384 PMC203906

[B23] Marsili E. Baron D. B. Shikhare I. D. Coursolle D. Gralnick J. A. Bond D. R. (2008). *Shewanella* secretes flavins that mediate extracellular electron transfer. *Proc. Natl. Acad. Sci. U.S.A.* 105 3968–3973. 10.1073/pnas.0710525105 18316736 PMC2268775

[B24] Meyer T. E. Tsapin A. I. Vandenberghe I. De Smet L. Frishman D. Nealson K. H. (2004). Identification of 42 possible cytochrome c genes in the *Shewanella oneidensis* genome and characterization of six soluble cytochromes. *J. Integr. Biol.* 8 57–77. 10.1089/153623104773547499 15107237

[B25] Myers J. D. Kelly D. J. (2005). A sulphite respiration system in the chemoheterotrophic human pathogen *Campylobacter jejuni*. *Microbiology* 151 233–242. 10.1099/mic.0.27573-0 15632441

[B26] Reguera G. McCarthy K. D. Mehta T. Nicoll J. S. Tuominen M. T. Lovley D. R. (2005). Extracellular electron transfer via microbial nanowires. *Nature* 435 1098–1101. 10.1038/nature03661 15973408

[B27] Richter K. Schicklberger M. Gescher J. (2012). Dissimilatory reduction of extracellular electron acceptors in anaerobic respiration. *Appl. Environ. Microbiol.* 78 913–921. 10.1128/AEM.06803-11 22179232 PMC3273014

[B28] Ringeisen B. R. Henderson E. Wu P. K. Pietron J. Ray R. Little B. (2006). High power density from a miniature microbial fuel cell using *Shewanella oneidensis* DSP10. *Environ. Sci. Technol.* 40 2629–2634. 10.1021/es052254w 16683602

[B29] Schuetz B. Schicklberger M. Kuermann J. Spormann A. M. Gescher J. (2009). Periplasmic electron transfer via the *c*-type cytochromes MtrA and FccA of *Shewanella oneidensis* MR-1. *Appl. Environ. Microbiol.* 75 7789–7796. 10.1128/AEM.01834-09 19837833 PMC2794085

[B30] Sturm G. Richter K. Doetsch A. Heide H. Louro R. O. Gescher J. (2015). A dynamic periplasmic electron transfer network enables respiratory flexibility beyond a thermodynamic regulatory regime. *ISME J.* 9 1802–1811. 10.1038/ismej.2014.264 25635641 PMC4511935

[B31] Summers Z. M. Fogarty H. E. Leang C. Franks A. E. Malvankar N. S. Lovley D. R. (2010). Direct exchange of electrons within aggregates of an evolved syntrophic coculture of anaerobic bacteria. *Science* 330 1413–1415. 10.1126/science.1196526 21127257

[B32] Ueki T. Nevin K. P. Rotaru A. E. Wang L. Y. Ward J. E. Woodard T. L. (2018). *Geobacter* strains expressing poorly conductive Pili reveal constraints on direct interspecies electron transfer mechanisms. *mBio* 9:e1273-18. 10.1128/mBio.01273-18 29991583 PMC6050967

[B33] Wang F. Gu Y. O’Brien J. P. Sophia M. Y. Yalcin S. E. Srikanth V. (2019). Structure of microbial nanowires reveals stacked hemes that transport electrons over micrometers. *Cell* 177 361–369. 10.1016/j.cell.2019.03.029 30951668 PMC6720112

[B34] Xiao X. Wu Y. Xu C. Cao D. Wang M. Ma X. (2012). Anaerobic respiratory capabilities of a metal-reducing microorganism *Shewanella* and its application in environmental remediation. *Microbiol. China* 39 1677–1686.

[B35] Xu M. Guo J. Cen Y. Zhong X. Cao W. Sun G. (2005). *Shewanella decolorationis* sp nov., a dye-decolorizing bacterium isolated from activated sludge of a waste-water treatment plant. *Int. J. Syst. Evol. Microbiol.* 55 363–368. 10.1099/ijs.0.63157-0 15653901

[B36] Yang Y. Xiang Y. Sun G. Wu W. M. Xu M. (2014). Electron acceptor-dependent respiratory and physiological stratifications in biofilms. *Environ. Sci. Technol.* 49 196–202. 10.1021/es504546g 25495895

[B37] Yang Y. Xu M. Guo J. Sun G. (2012). Bacterial extracellular electron transfer in bioelectrochemical systems. *Process Biochem.* 47 1707–1714. 10.1016/j.procbio.2012.07.032

[B38] Yong Y. C. Yu Y. Y. Yang Y. Li C. M. Jiang R. Wang X. (2012). Increasing intracellular releasable electrons dramatically enhances biocurrent output in microbial fuel cells. *Electrochem. Commun.* 19 13–16. 10.1016/j.elecom.2012.03.002

[B39] Zhang T. Bain T. S. Barlett M. A. Dar S. A. Snoeyenbos-West O. L. Nevin K. P. (2014). Sulfur oxidation to sulfate coupled with electron transfer to electrodes by *Desulfuromonas* strain TZ1. *Microbiology* 160 123–129. 10.1099/mic.0.069930-0 24169815

