# Supplementary material for: Lack of Periplasmic Non-heme Protein SorA Increases Shewanella decolorationis Current Generation
Source: Front Microbiol. 2020 Feb 25;11:262. doi: 10.3389/fmicb.2020.00262 (PMC7052111; doi:10.3389/fmicb.2020.00262)
Supplement: Supplementary file 1 [file Data_Sheet_1.docx]

**Supplementary materials**

**Table S1.** Expression of genes whose FPKM log_2_ fold change is greater than two in electrode respiration compared to that in azo reduction.

| Gene ID | Gene | Product | FPKM  Azo-dye | FPKM  MFC | Log2 fold change | FDR  p-value |
| --- | --- | --- | --- | --- | --- | --- |
| SHD_0058 | SHD0058 | dependent receptor | 60.316 | 452.399 | 2.907 | 4.32E-80 |
| SHD_0282 | SHD0282 | phage integrase family protein | 0.094 | 0.472 | 2.328 | 8.17E-02 |
| SHD_0455 | SHD0455 | methyl-accepting chemotaxis protein | 28.978 | 171.575 | 2.566 | 1.58E-65 |
| SHD_0492 | SHD0492 | hypothetical protein | 27.082 | 1276.248 | 5.558 | 5.96E-247 |
| SHD_0493 | SHD0493 | amp-binding protein | 31.465 | 163.02 | 2.373 | 2.65E-26 |
| SHD_0539 | SHD0539 | hypothetical protein | 362.782 | 1489.183 | 2.037 | 4.13E-28 |
| SHD_0573 | SHD0573 | n-acetylglutamate synthase | 66.4 | 313.918 | 2.241 | 9.88E-48 |
| SHD_0598 | SHD0598 | bacteriophage replication protein | 290.077 | 1171.996 | 2.014 | 3.53E-53 |
| SHD_0679 | SHD0679 | MltA-interacting MipA family protein | 166.761 | 720.544 | 2.111 | 1.86E-62 |
| SHD_1001 | SHD1001 | exported protein | 26.333 | 981.925 | 5.221 | 3.77E-57 |
| SHD_1325 | argC | n-acetyl-gamma-glutamyl-phosphate reductase | 84.292 | 353.041 | 2.066 | 4.23E-48 |
| SHD_1385 | SHD1385 | bifunctional autotransporter extracellular effector | 20.041 | 1956.325 | 6.609 | 5.03E-140 |
| SHD_1386 | SHD1386 | propeptide amd peptidase m4 | 89.458 | 817.907 | 3.193 | 4.37E-85 |
| SHD_1387 | phoP-2 | two component transcriptional winged helix family | 72.088 | 860.602 | 3.578 | 3.09E-151 |
| SHD_1388 | phoQ-1 | integral membrane sensor signal transduction histidine kinase | 26.867 | 327.333 | 3.607 | 2.45E-86 |
| SHD_1389 | phoQ-2 | integral membrane sensor signal transduction histidine kinase | 31.588 | 326.388 | 3.369 | 4.37E-85 |
| SHD_1882 | SHD1882 | ornithine decarboxylase | 73.691 | 921.121 | 3.644 | 5.13E-154 |
| SHD_2017 | nhaC-2 | na+/h+ antiporter | 138.218 | 588.566 | 2.090 | 1.55E-59 |
| SHD_2293 | fdhD-3 | formate dehydrogenase accessory protein | 12.183 | 87.034 | 2.837 | 9.23E-20 |
| SHD_2294 | SHD2294 | oxidoreductase alpha subunit | 19.819 | 194.729 | 3.297 | 4.27E-44 |
| SHD_2425 | phhA | phenylalanine 4-monooxygenase | 141.154 | 684.317 | 2.277 | 3.09E-73 |
| SHD_2426 | phhB | pterin-4-alpha-carbinolamine dehydratase | 95.242 | 433.545 | 2.187 | 2.55E-69 |
| SHD_2781 | SHD2781 | secreted protein | 36.506 | 259.372 | 2.829 | 1.66E-57 |
| **SHD_2782** | **mcc** | **monoheme cytochrome c** | **12.913** | **262.978** | **4.348** | **1.41E-51** |
| **SHD_2783** | **sorB** | **monoheme cytochrome** | **23.716** | **186.698** | **2.977** | **8.04E-19** |
| **SHD_2784** | **sorA** | **molybdopterin containing oxidoreductase** | **38.259** | **537.729** | **3.813** | **8.62E-63** |
| **SHD_2785** | **SHD2785** | **monoheme cytochrome c** | **23.1** | **391.766** | **4.084** | **2.84E-68** |
| SHD_3489 | SHD3489 | wd-40 repeat-containing protein | 46.674 | 226.929 | 2.282 | 5.25E-78 |
| SHD_3585 | SHD3585 | serine endoprotease | 79.834 | 431.812 | 2.435 | 1.47E-74 |
| SHD_3628 | SHD3628 | gtp pyrophosphokinase | 51.016 | 413.013 | 3.017 | 8.68E-133 |
| SHD­_3778 | SHD3778 | hypothetical protein | 66.002 | 2393.536 | 5.180 | 1.13E-220 |
| SHD_3891 | SHD3891 | RND family efflux transporter MFP subunit | 270.442 | 1929.442 | 2.835 | 1.55E-72 |
| SHD_3892 | SHD3892 | acriflavin resistance protein | 177.425 | 1329.2 | 2.905 | 4.64E-88 |
| SHD_3940 | SHD3940 | hypothetical protein | 3.137 | 15.552 | 2.310 | 3.22E-06 |
| SHD_4005 | SHD4005 | EmrB/QacA drug resistance subfamily | 65.653 | 681.458 | 3.376 | 1.08E-123 |
| SHD_4006 | SHD4006 | secretion protein family protein | 153.547 | 1569.51 | 3.354 | 4.32E-80 |

FPKM: Fragments per kilobase per million mapped reads. FDR: false discovery rate

**Table S2.** Distribution of gene cluster similar to *SHD2782-2785 in Shewanella* genomes.

| **Description** | **Per. Ident** | **Gene accession** |
| --- | --- | --- |
| *Shewanella decolorationis* strain sesselensis | 98.93% | CP037898.1 |
| *Shewanella decolorationis* strain Ni1-3 | 98.84% | CP031775.1 |
| *Shewanella* sp. FDAARGOS_354 | 96.13% | CP022089.2 |
| *Shewanella oneidensis* MR-1, | 95.77% | AE014299.2 |
| *Shewanella* sp. Pdp11 | 83.63% | CP015194.1 |
| *Shewanella baltica* BA175 | 82.34% | CP002767.1 |
| *Shewanella* sp. WE21 | 82.28% | CP023019.1 |
| *Shewanella putrefaciens* strain NCTC10737 | 81.68% | LR134321.1 |
| *Shewanella baltica* OS117, | 81.68% | CP002811.1 |
| *Shewanella baltica* OS155 | 81.68% | CP000563.1 |
| *Shewanella baltica* strain CW2 | 81.31% | CP028355.1 |
| *Shewanella baltica* OS678 | 81.28% | CP002383.1 |
| *Shewanella baltica* OS195 | 81.28% | CP000891.1 |
| *Shewanella baltica* strain 128 | 80.75% | CP028730.1 |


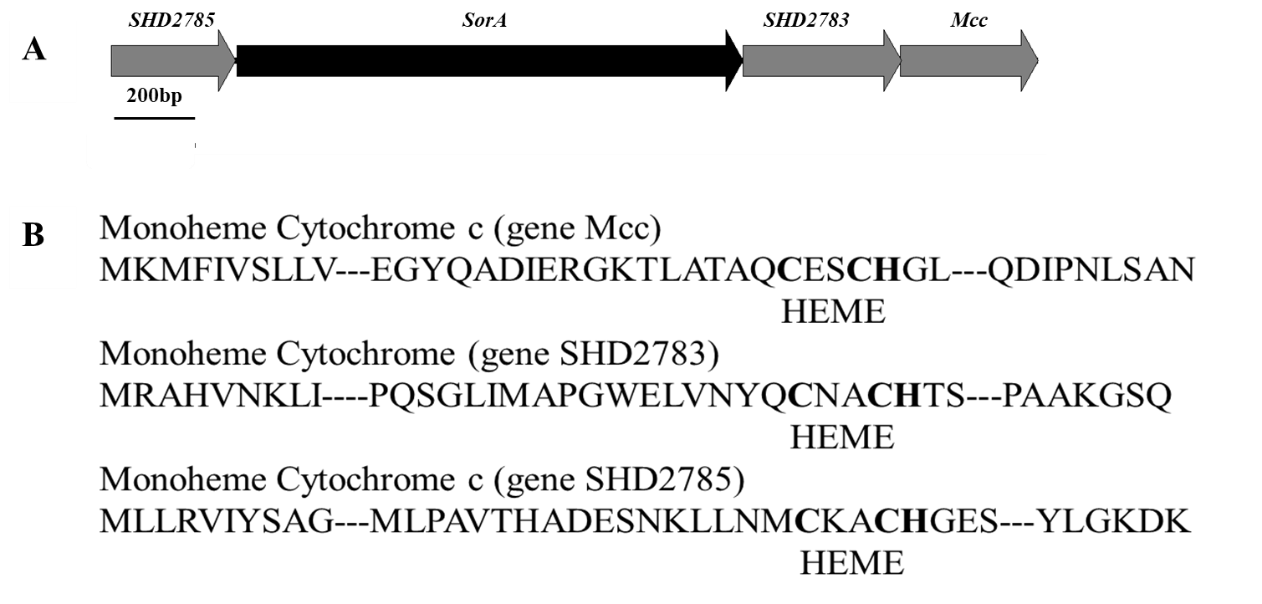


**Figure S1.** SHD2782-2785 gene and protein sequence analysis. (A) Gene arrangement map. (B) CXXCH structure in protein sequences.





**Figure S2.** CV of the LM medium, mutant strain Δ*mtrC&omcA* and the mix flavins of riboflavin and flavin mononucleotide.


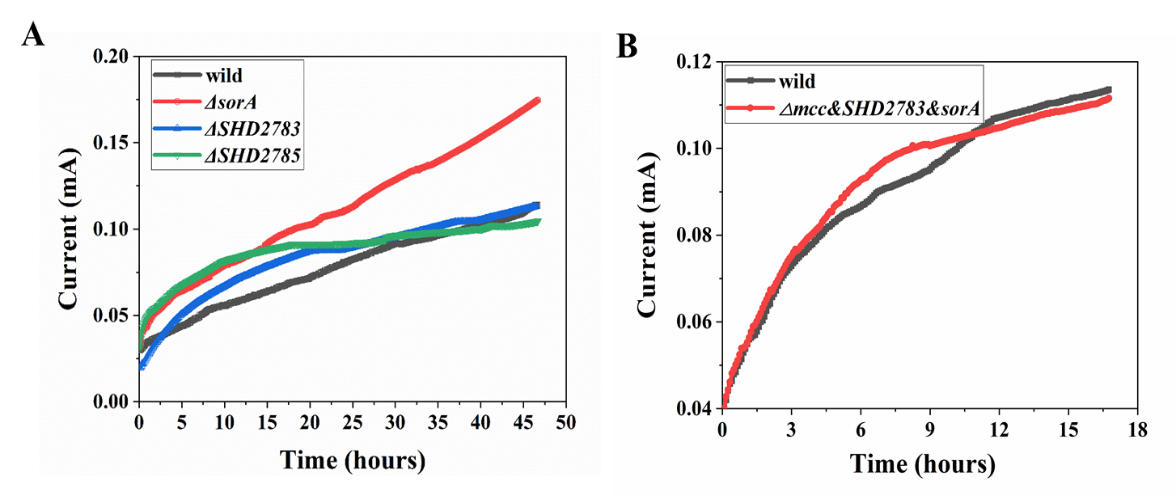


**Figure S3.** (A) Electricity generation of mutant strain Δ*SHD2783*, Δ*sorA*, Δ*SHD2785* under microbial fuel cell system. (B) Electricity generation of multi-mutant strain Δ*mcc&SHD2783&sorA* in microbial fuel cells.


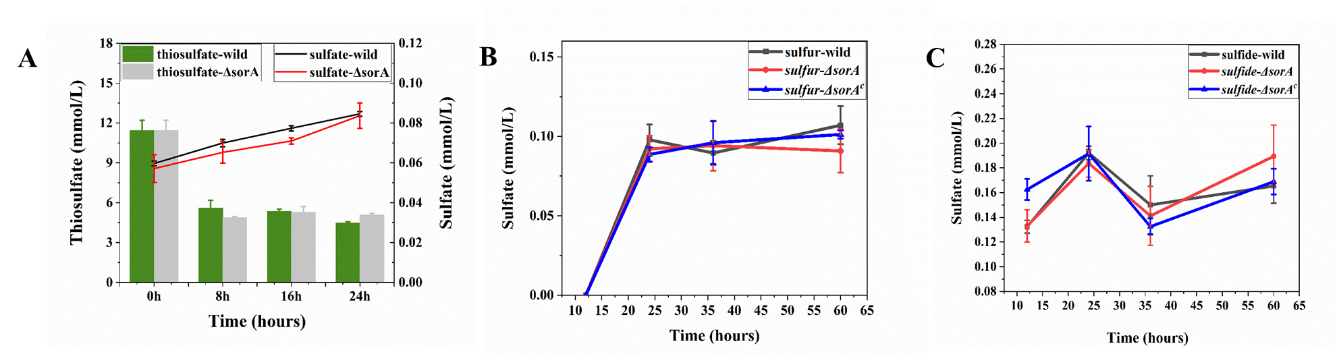


**Figure S4. (A)** Sulfate generation during thiosulfate oxidation of Δ*sorA*, wild strain**. (B)** Sulfate generation during sulfur oxidation of Δ*sorA*, wild strain and complementary strain**. (C)** Sulfate generation during sodium sulfide oxidation of Δ*sorA*, wild strain and complementary strain**.**


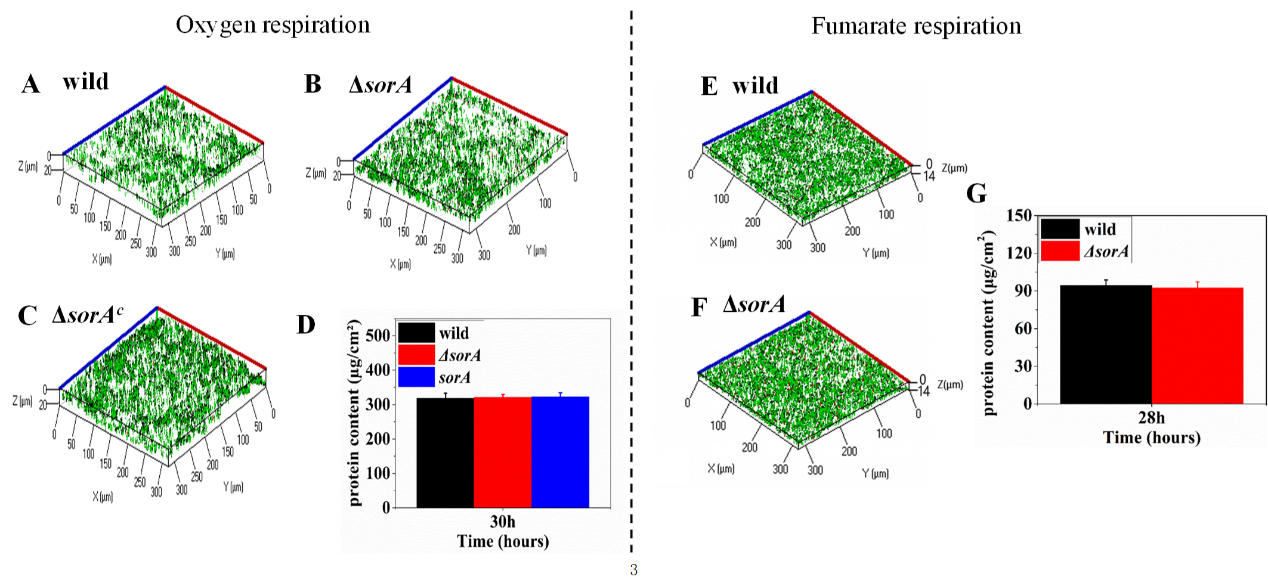


**Figure S5.** (A-C) Biofilms of wild, mutant and complemented strain under aerobic condition. (D) Protein content on the graphite plate of the wild, mutant and complemented strain under aerobic condition. (E, F) Biofilms of wild and mutant strain respiring with fumarate. (G) Protein content on the graphite plate of the wild and mutant strain respiring with fumarate.
